# Highly efficient hairy root genetic transformation and applications in citrus

**DOI:** 10.3389/fpls.2022.1039094

**Published:** 2022-10-27

**Authors:** Haijie Ma, Xinyue Meng, Kai Xu, Min Li, Fred G. Gmitter, Ningge Liu, Yunpeng Gai, Suya Huang, Min Wang, Min Wang, Nian Wang, Hairen Xu, Jinhua Liu, Xuepeng Sun, Shuo Duan

**Affiliations:** ^1^ Collaborative Innovation Center for Efficient and Green Production of Agriculture in Mountainous Areas of Zhejiang Province, College of Horticulture Science, Zhejiang A&F University, Hangzhou, Zhejiang, China; ^2^ China-USA Citrus Huanglongbing Joint Laboratory (A Joint Laboratory of The University of Florida’s Institute of Food and Agricultural Sciences and Gannan Normal University), National Navel Orange Engineering Research Center, Gannan Normal University, Ganzhou, Jiangxi, China; ^3^ Citrus Research and Education Center, Horticultural Sciences Department, University of Florida, Lake Alfred, FL, United States; ^4^ School of Grassland Science, Beijing Forestry University, Beijing, China; ^5^ Natural Medicine Institute of Zhejiang YangShengTang Co., LTD, Hangzhou, Zhejiang, China

**Keywords:** citrus, genetic transformation, genome editing, *Agrobacterium rhizogenes*, virus

## Abstract

Highly efficient genetic transformation technology is greatly beneficial for crop gene function analysis and precision breeding. However, the most commonly used genetic transformation technology for woody plants, mediated by *Agrobacterium tumefaciens*, is time-consuming and inefficient, which limits its utility for gene function analysis. In this study, a simple, universal, and highly efficient genetic transformation technology mediated by *A. rhizogenes* K599 is described. This technology can be applied to multiple citrus genotypes, and only 2–8 weeks were required for the entire workflow. Genome-editing experiments were simultaneously conducted using 11 plasmids targeting different genomic positions and all corresponding transformants with the target knocked out were obtained, indicating that *A. rhizogenes*-mediated genome editing was highly efficient. In addition, the technology is advantageous for investigation of specific genes (such as *ACD2*) for obtaining “hard-to-get” transgenic root tissue. Furthermore, *A. rhizogenes* can be used for direct viral vector inoculation on citrus bypassing the requirement for virion enrichment in tobacco, which facilitates virus-induced gene silencing and virus-mediated gene expression. In summary, we established a highly efficient genetic transformation technology bypassing tissue culture in citrus that can be used for genome editing, gene overexpression, and virus-mediated gene function analysis. We anticipate that by reducing the cost, required workload, experimental period, and other technical obstacles, this genetic transformation technology will be a valuable tool for routine investigation of endogenous and exogenous genes in citrus.

## Introduction

Citrus is among the most important fruit crops worldwide and is grown in more than 114 countries (Talon and Gmitter, 2008). Global predicted citrus production exceeded 146 million tons (FAOSTAT; https://www.fao.org/faostat/en). The citrus industry currently requires new cultivars with desirable traits to improve yields, nutritional value, and adaptability to biotic and abiotic stresses. The application of genetic transformation to improve citrus has increased in recent years (Peña, 2000; Boscariol et al., 2006; Fagoaga et al., 2006; Febres et al., 2008; Gambino and Gribaudo, 2012). *Agrobacterium*-mediated transformation of epicotyl segments requires tissue culture, which is widely employed to produce disease-resistant materials in the laboratory, remains the quickest method for improvement of citrus cultivars. To date, many important traits have been successfully introduced into different citrus species and hybrids, such as lime, sweet orange, and grapefruit (Domínguez et al., 2002; Fu et al., 2011; Jia et al., 2017; Peng et al., 2017; Jia et al., 2021). However, transformation mediated by *Agrobacterium tumefaciens* has many disadvantages, including that the procedure is time consuming, laborious, expensive, and inefficient. The low frequency of rooting is an additional limitation of *A. tumefaciens*-mediated transformation of epicotyl segments of citrus (Gutiérrez-E. et al., 1997). As a result, micrografting is frequently used for maintenance of transgenic plants (Poles et al., 2020). Therefore, a rapid and highly efficient genetic transformation method bypassing the need for tissue culture is critical for gene function analysis and genetic improvement of citrus.

*Agrobacterium* species are widely used to generate transgenic plants as the agrobacteria can integrate transfer DNA (T-DNA) into a host plant’s nuclear DNA genome. *Agrobacterium tumefaciens* transfers the tumor-inducing (Ti) plasmid into the host nucleus to incorporate exogenous DNA into a host chromosome and subsequently cause formation of a tumor at the plant wound site. This mechanism has been utilized for *A. tumefaciens*-mediated plant transformation of many plant species to improve crop traits and for research on gene function (Chetty et al., 2013; Kaur and Sah, 2014). In recent decades, *A. tumefaciens* has been widely applied in citrus breeding and gene functional research (Domínguez et al., 2002; Stover et al., 2013; Hongge et al., 2019). However, for most plants, especially woody species, when using *A. tumefaciens*, the generation of stable transformants requires plant regeneration from a few cells or even a single cell using exogenous phytohormones, and thus the process is time consuming and laborious. In addition, *Agrobacterium rhizogenes* has been successfully used in plant genetic transformation technologies (Estrada-Navarrete et al., 2007). *Agrobacterium rhizogenes* can infect plants to induce formation of hairy roots from wounded tissue owing to the expression of *rol* genes encoded in the Ri plasmid. The T-DNA cassette from the exogenous binary vector can be transferred and integrated into the host cell genome together with T-DNA from the Ri plasmid (White et al., 1985). Compared with *A. tumefaciens*, *A. rhizogenes*-mediated hairy root genetic transformation technology bypassing the requirement for tissue culture and antibiotic screening is highly efficient and has been widely used in many herbaceous plants for rhizosphere physiology research and recombinant protein production (Tsuro et al., 2005; Majumdar et al., 2011; Habibi et al., 2016; Beigmohamadi et al., 2019). However, *A. rhizogenes*-mediated genetic transformation in woody plants bypassing tissue culture remains at an early stage of application (Irigoyen et al., 2020).

In this study, we describe a rapid and highly efficient root genetic transformation and genome-editing protocol for citrus using *A. rhizogenes* strain K599. This technique requires only 2–8 weeks for completion, bypasses tissue culture, and has applicability for diverse citrus accessions. To date, no report is available on a highly efficient endogenous gene-editing technology for citrus that bypasses tissue culture. In addition, the proposed protocol can be used for viral vector inoculation bypassing tobacco-mediated virion enrichment, which can improve the efficiency of virus-mediated analysis of citrus gene function. We anticipate that the protocol will be a valuable tool for routine investigation of endogenous and exogenous genes in citrus.

## Materials and methods

### Bacterial strains, plant material, and growth conditions

The *Escherichia coli* (DH5α) competent cells (CAT#: DL1002), *A. rhizogenes* (K599) competent cells (CAT#: AC1080), and *A. tumefaciens* (EHA105) competent cells (CAT#: AC1012) were obtained from Shanghai Weidi Biotechnology Co., Ltd. All transformed bacterial strains were stored in 15% glycerol and preserved in a freezer at −80°C. *Escherichia coli* cells were cultured in lysogeny broth medium at 37°C. The K599 and EHA105 strains were recovered and cultured at 28°C in tryptone yeast medium with corresponding antibiotics.

Branches of *Citrus medica*, *C. limon*, *C. sinensis*, and citrange ’Carrizo’ were obtained from the National Citrus Engineering Research Center, Chongqing, China. Plants with transgenic hairy root were grown in a greenhouse at 26°C with a 16 h/8 h (light/dark) photoperiod. All citrus plants were cultured in a net greenhouse under natural conditions.

### Agrobacterium-infiltrated citrus hairy root transformation

Recombinant *A. rhizogenes* strains were cultured in fresh yeast extract peptone medium with appropriate antibiotics at 28°C. The resuspended *A. rhizogenes* K599 cells at the final concentration (OD_600_ = 0.6) were diluted into the MES solution (10 mM MgCl_2_, 10 mM MES [pH 5.6], and 200 µM AS). Blade-removed citrus branches (approximately 2 months old) were collected from the greenhouse. We cut the stems into ~10 cm sections using sterilized shears by keeping the smooth surface of the cross-section.

The base of the stems sections was soaked in the *A. rhizogenes* K599 suspension and vacuum infiltrated for approximately 25 min using a standard vacuum. The stem sections were cultured in a dome tray filled with vermiculite-mixed soil in the greenhouse at 26°C with 90% relative humidity and a 16 h/8 h (light/dark) photoperiod. Hairy root development began after approximately 2–4 weeks (*C. medica*) or 4–8 weeks (*C. limon* and citrange ‘Carrizo’) after agroinfiltrated transformation. Potential transgenic roots were detected by the fluorescence signal with a portable excitation lamp (Luyor-3415RG, Shanghai, China). The fluorescence-positive hairy roots were incubated in liquid modified Hoagland’s nutrient medium (Coolaber, Beijing, China) in sterile tubes for observation of symptoms. Transgenic roots were cultured at 26°C under a 16 h/8 h (light/dark) photoperiod. The symptoms were captured with a digital camera (Canon EOS 200D, Tokyo, Japan). Those shoots were further confirmed by PCR analysis. The hairy root transformation efficiency was calculated using the following formula: [(Number of GFP-containing roots)/(Total number of roots)] × 100. The GFP fluorescence in transgenic citrus roots was observed with a confocal microscope (LSM 780, Carl Zeiss, Jena, Germany) with 488 nm excitation and 505–530 nm emission wavelengths.

### DNA and RNA extraction

Genomic DNA from roots was extracted using the cetyltrimethylammonium bromide method (Richards et al., 1994). Total RNA from the root and callus was extracted using the RNA Isolater Total RNA Extraction Reagent (R401-01, Vazyme, Nanjing, China). Gel electrophoresis and a NanoDrop spectrophotometer (NanoDrop Technologies, Inc., Wilmington, DE, USA) were used to assess RNA quantity and quality. The cDNA was synthesized using the HiScript III 1st Strand cDNA Synthesis Kit (+gDNA wiper) (R312-01, Vazyme). RT-qPCR analysis was conducted using the AceQ Universal SYBR qPCR Master Mix (Q511-02, Vazyme) and a real-time PCR system (Q2000A, LongGene, Hangzhou, China). The internal control gene used was actin and the corresponding primers were listed in **Table S1**.

### Transcriptome analysis

The RNA-sequencing experiments were conducted using three biological replicates of each sample. Sequencing libraries were generated using the NEBNext Ultra II RNA Library Prep Kit (New England Biolabs, Ipswich, MA, USA) and were sequenced on an Illumina NovaSeq 6000 Sequencing System in paired-end mode. The raw reads were processed with Trimmomatic v. 0.36 (Bolger et al., 2014) to remove adaptor sequences and low-quality reads. The cleaned reads were aligned to the Citrus *medica* genome using HISAT2 v. 2.2.1 (Kim et al., 2015). The number of reads mapped to each gene was counted with htseq-count v. 1.99.2. Differential expression between the C. *medica* wild type and C. *medica* agroinfiltrated explants was analyzed using the ‘DESeq2’ R package (Love et al., 2014). Genes with an adjusted *p*-value (*p*_adj_ ≤ 0.05) and at least two-fold change in expression were assigned as DEGs. To explore the functions and pathways of the DEGs, GO terms and KEGG pathway enrichment analyses were performed using the ‘ClusterProfiler’ R package (Wu et al., 2021) and were visualized using the ‘ggplot2’ R package. The selected KEGG pathways associated with “plant hormone signal transduction”, “amino acid biosynthesis”, “plant–pathogen interaction”, and “MAPK signaling pathway” were visualized using the ‘Pathview’ R package (Weijun and Cory).

### Sequencing analysis

All transgenic roots and the wild-type plants were subjected to PCR (P111-01, Vazyme) using gene-specific primers (**Table S1**) to amplify DNA insertions or fragments including the target sites. The PCR amplicons were cloned into the pGEM^®^-T Easy vector (Promega, Madison, WI, USA) for Sanger sequencing. The sequence chromatograms were analyzed with SnapGene software.

### Visualization of GFP fluorescent signal

Transgenic roots were first confirmed with a hand-held excitation lamp (Luyor-3415RG). For microscopic inspection, roots were rinsed with ddH_2_O and photographed under a Leica-M205FA stereomicroscope (Leica Microsystems, Wetzlar, Germany). Two different autofluorescence emission wavelength bands were used for detection: green (505–550 nm) and red (>560 nm), defined by optical filters. Transverse sections of the transgenic roots were cut with a razor blade as thinly as possible. The sections were mounted on a glass microscope slide in ddH_2_O. The GFP signal was observed under a Leica-SP8MP confocal fluorescence microscope (Leica Microsystems). The fluorescence was observed under excitation at 488 nm and emission at 505–550 nm. Simultaneously, images (1920 × 1024 pixels) were captured with a suitable scale bar.

### β-Glucuronidase expression

The GUS (*uidA*) gene expression was detected using a GUS gene quantitative detection kit (SL7161, Coolaber) following the manufacturer’s instructions. Briefly, roots and hairy roots were incubated in 0.1 M sodium phosphate buffer with GUS substrate for 12 h at 37°C. The enzymatic reaction was stopped with 70% ethanol. Tissues were observed with a light stereomicroscope after GUS staining.

### GRNA design

sgRNAs applied in this study were designed using the online tools CRISPRP v2.0 (http://crispr.hzau.edu.cn/cgi-bin/CRISPR2/CRISPR) based on their evaluation score (Rank from high to low), GC content (40%-60%) and putative off-target sites (Rank from low to high). target sequences with 20-bp were designed for each gene in the first exon. The purpose of this design will increase the possibility of affecting protein function and the likelihood that at least one site would be edited.

### Virus inoculation

Prepare the *A. rhizogene* strain K599 harboring corresponding CLBV-based vector. Grow the corresponding *A. rhizogene* strain in YEP medium till OD_600_ = 0.8. Spin down the pellets and wash twice with infiltration medium (MES medium: 10 mM MES, 10 mM MgCl_2_, 200 µm acetosyringone). Re-suspend the pellets using infiltration medium till OD_600_ = 0.5. Place the infiltration medium at 25°C for 3 hours in dark condition. Conduct vacuum agroinfiltration using citrus explants and maintain explants in greenhouse at 25°C. Observe the phenotypes after several months and confirm the transcription of target genes by RT-qPCR. All the experiments were conducted using at least three replicates.

### Data availability statement

All datasets supporting the conclusions of this article are included in the article and supplementary files. The transcriptome project has been deposited at NCBI BioProject under the accession PRJNA800116 (https://www.ncbi.nlm.nih.gov/bioproject/PRJNA800116). All gene sequences, genome, gff3 file, and proteomes, gene ontology (GO), COG category, CAZy, KEGG, PFAMs, NR, eggNOG annotation of C. medica for bioinformatic analysis are available in the Zenodo repository at https://doi.org/10.5281/zenodo.5902607. The high-resolution figures are available on the figshare repository: https://doi.org/10.6084/m9.figshare.19060802.v1. The raw sequence data used for transcriptome analysis are available in NCBI under the Sequence Read Archive (SRA) with the SRA accession number for *C. medica* GFP agroinfiltrated explants: SRR17731820, SRR17731819, SRR17731818; *C. medica* wild-type: SRR17731817, SRR17731816, SRR17731815.

## Results

### Explants survival of different citrus genotypes in vermiculite

**Figure 1:**
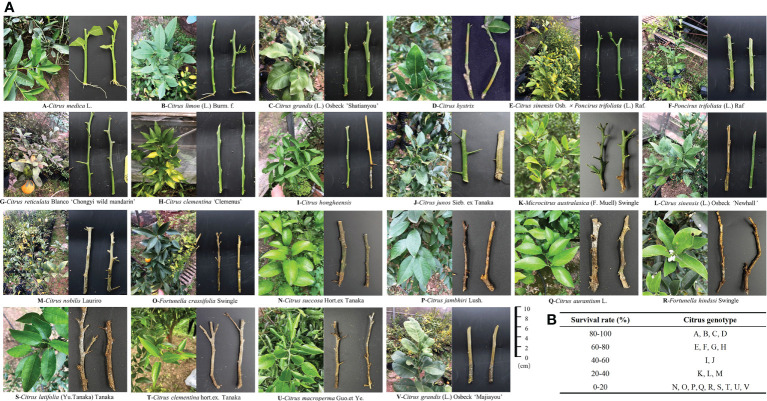
Comparative analysis of percentage explant survival of citrus accessions in vermiculite. **(A)** Citrus explants were incubated in vermiculite [28°C, 16 h/8 h (light/dark), and 90% relative humidity] after vacuum infiltration. Statistical analysis was performed on the survival data for explants at 15, 30 and 45 days post-infiltration (dpi), respectively. **(B)** Statistical analysis of explant survival.

*Agrobacterium rhizogenes* integrates T-DNA from the Ri plasmid into the host plant genome when sensing signal substances, such as acetosyringone (AS), so as to induce formation of hairy roots and to synthesize substances needed for growth of the bacteria **(** **Figure S1** **)**. To achieve genetic transformation bypassing tissue culture, the percentage explant survival of 22 citrus genotypes in vermiculite was first analyzed, ranging from 0% to 95% at 45 days post-incubation **(** **Table S2** **)**. The four citrus genotypes with the highest percentage survival (>80%) were *Citrus medica*, *C. limon*, *C. grandis* ‘Shatianyou’, and *C. hystrix*. **(** **Figure 1A** **)**. Most branches of these four genotypes eventually produced roots for more than 10 genotypes, the percentage survival was less than 40% within 45 days and most of the branches failed to produce roots **(** **Figure 1B** **)**.

### K599-mediated genetic transformation of citrus bypassing tissue culture

**Figure 2:**
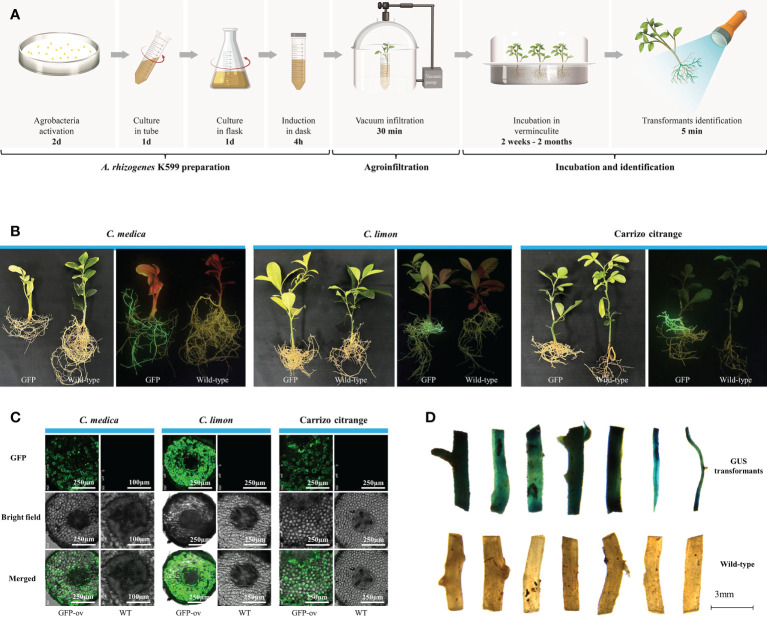
*Agrobacterium rhizogenes* K599 mediated highly efficient genetic transformation bypassing tissue culture. **(A)** Schematic diagram of the protocol for conducting A. *rhizogenes*-mediated genetic transformation bypassing tissue culture. **(B)** Images of *Citrus medica*, *C*. *limon*, and citrange ‘Carrizo’ at 4 months post-agroinfiltration. GFP, citrus explants infiltrated with K599 harboring a binary plasmid containing *GFP*; Wild-type, untreated citrus explants. The left images were taken under white light, and the right images were taken under excitation light. **(C)** Green fluorescent protein (GFP) signal visualized by laser scanning confocal microscopy in transgenic tissues of citrus roots. GFP, green fluorescent protein expression signal observed with a Leica-SP8MP confocal fluorescence microscope (excitation 488 nm, emission 505–550 nm); Bright, brightfield; Merged, merged GFP and brightfield images. **(D)** GUS staining of roots.

Based on the percentage survival, we selected *C. medica*, *C. limon*, and citrange ‘Carrizo’ for assessment of the efficiency of genetic transformation of citrus branches mediated by *A. rhizogenes* K599 harboring a binary plasmid (1380-Cas9-HA) overexpressing *GFP* **(** **Figure S2**; **Table S3** **)**. Briefly, the genetic transformation protocol comprised three steps: K599 and explant preparation, vacuum infiltration, and explant incubation in vermiculite **(** **Figure 2A**; **Table S4** **)**. The fluorescent transgenic hairy roots began to develop 2 weeks (*C. medica*) or 4 weeks (*C. limon* and citrange ‘Carrizo’) post-incubation in vermiculite **(** **Figure S3** **)**. The length and number of fluorescent hairy roots increased significantly after 4 months **(** **Figure 2B** **)**. Non-transgenic hairy roots lacking GFP signal also emerged, but these roots had no impact on subsequent research as they were easily distinguishable based on GFP fluorescence and were readily removed. Confocal microscopic examination confirmed that GFP fluorescence was universally distributed in transgenic hairy roots but was not observed in non-transgenic hairy roots **(** **Figure 2C** **)**. Subsequently, genetic transformation in citrange ‘Carrizo’ was performed using a different binary plasmid carrying a gene encoding protein β-glucuronidase (GUS) **(** **Table S3** **)**. Staining of GUS revealed that dark blue-stained transgenic hairy roots were induced by K599 harboring the corresponding plasmid, whereas no GUS staining was detected in the control **(** **Figure 2D** **)**. The transformation rate of corresponding plasmids or citrus species was listed in **Table S5**. Compared with *A. tumefaciens*-mediated citrus genetic transformation, which usually takes 3–6 months with a low success rate, the K599-mediated genetic transformation method was time-saving and cost-effective.

### K599-mediated genetic transformation and genome editing is highly efficient

**Figure 3:**
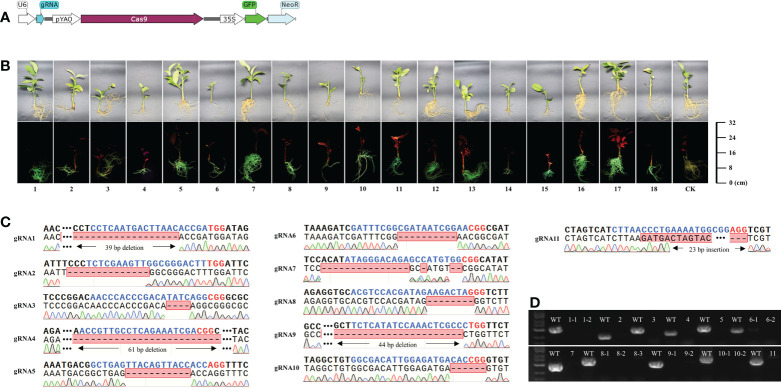
*Agrobacterium rhizogenes* K599-mediated genetic transformation and genome editing is highly efficient. **(A)** Schematic diagrams of genome-editing elements used in the study. **(B)** Green fluorescence signal visualized by laser scanning confocal microscopy in transgenic tissues of citrus roots. **(C)** Alignment of the genome-edited sequence of corresponding transgenic hairy roots with the gRNA-targeted loci. Blue nucleotides indicate gRNA and red nucleotides indicate PAM. **(D)** PCR assays conducted at gRNA-targeted sites of transgenic and wild-type roots.

To verify the efficiency of K599-mediated genetic transformation and genome editing, transformation was performed using 18 transformed K599 strains, each containing a different plasmid. All 18 plasmids carried the *GFP* and *Neo* encoding genes, and 11 contained genome-editing elements targeting different loci in the citrus genome **(** **Figure 3A**; **Figure S4**; **Tables S3** and **S6** **)**. Branches of *C. medica* were vacuum infiltrated using these transformed K599 strains. After one month, transgenic hairy roots corresponding to these 18 plasmids were obtained. Owing to the presence of the *GFP* selection marker, non-transgenic hairy roots were easily detected and removed, and thus *C. medica* with only transgenic hairy roots was obtained **(** **Figure 3B** **)**. The *GFP* and *Neo* genes were both amplified from genomic DNA isolated from the transgenic hairy roots **(** **Figure S5** **)**. Sequencing revealed that the gRNA target in the corresponding transgenic hairy roots had been successfully edited **(** **Figure 3C** **)**. The missing nucleotides in edited transformants were mainly located at the 5’ end of the PAM site, which is consistent with the properties of Cas9-mediated DNA cleavage. To detect possible chimeras in the transgenic hairy root tissue, primers **(** **Table S1** **)** were designed to amplify the edited loci based on the sequencing results. No fragments were amplified by PCR from the edited hairy roots, and specific fragments of the expected length were amplified from wild-type explants **(** **Figure 3D** **)**, confirming the absence of chimeras in the edited hairy roots. Furthermore, PCR amplification and sequencing were performed on one randomly selected edited hairy root (gRNA10) using primers that flanked the gRNA-targeted locus **(** **Table S1** **)**. A total of 20 random colonies were sequenced, which all contained changed sequences at the gRNA targeted locus, further confirming that the tested transformant was not a chimera **(** **Figure S6** **)**.

### *Agrobacterium rhizogenes* significantly affects expression of genes involved in hormone signal transduction

**Figure 4:**
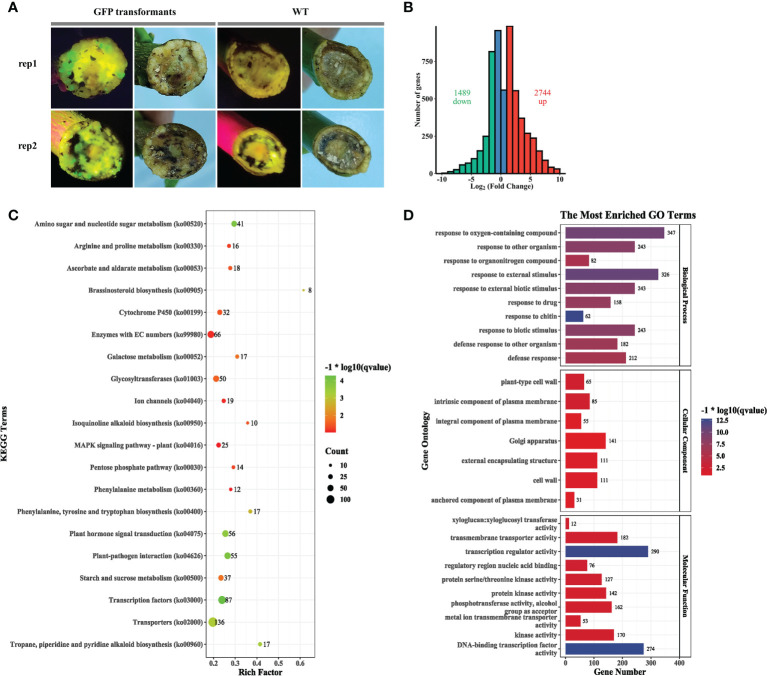
*Agrobacterium rhizogenes* K599 significantly affects expression of genes involved in hormone signal transduction. **(A)** Phenotype of callus generated on the wound surface of explants 2 weeks post-agroinfiltration. **(B)** Volcano plot (left) and histogram (right) of callus 2 weeks post-agroinfiltration compared with the wild type. **(C)** KEGG pathway analysis of differentially expressed genes (DEGs). The count represents the numbers of DEGs annotated for the pathway term. The *q* value is the adjusted *p* value. **(D)** Gene ontology (GO) enrichment analysis of DEGs. The results are summarized for the three GO categories (cellular component, molecular function, and biological process). The *x*-axis represents the number of DEGs in a category; the *y*-axis represents the GO term.

Compared with the wild-type explant lacking fluorescence, several GFP fluorescence spots were observed in callus formed at the wound surface of *C. medica* explants at 10 days post-agroinfiltration **(** **Figure 4A** **)**, indicating that K599 successfully transferred the binary plasmid into numerous cells. Transcriptome sequencing (PRJNA800116) of callus from wild-type and agroinfiltrated explants was conducted. A total of 4,233 differentially expressed genes (DEGs), comprising 2,744 upregulated and 1,489 downregulated genes, were identified in agroinfiltrated tissues compared with the non-treated control **(** **Figure 4B**; **Table S7** **)**. Kyoto Encyclopedia of Genes and Genomes (KEGG) pathway enrichment analysis revealed that many DEGs in agroinfiltrated tissue were enriched in the “transcription factors” (87), “plant–pathogen interaction (55)”, “amino sugar and nucleotide sugar metabolism” (41), “plant hormone signal transduction” (56), “transporters” (136), “starch and sucrose metabolism” (37), “glycosyltransferases” (50), “cytochrome P450” (32), and “enzymes with EC numbers” (66) **(** **Figure 4C**; **Table S8** **)**. Gene ontology (GO) enrichment analysis revealed that many DEGs in agroinfiltrated tissue were enriched in “DNA-binding transcription factor activity” (274), “transcription regulator activity” (290), “response to external stimulus” (326), “response to oxygen-containing compound” (347), “response to other organism” (243), “response to external biotic stimulus” (243), “response to biotic stimulus (243), and “defense response” (212) **(** **Figure 4D**; **Table S9** **)**. Further analysis revealed that many DEGs enriched in the pathways “Plant hormone signal transduction” **(** **Figure S7** **)**, “Phenylalanine, tyrosine and tryptophan biosynthesis” **(** **Figure S8** **)**, “Plant–pathogen interaction” **(** **Figure S9** **)**, and “MAPK signaling pathway” **(** **Figure S10** **)** were upregulated.

### *Agrobacterium rhizogenes*-mediated genetic transformation complements “hard-to-get” transgenic materials and facilitates gene function analysis

**Figure 5:**
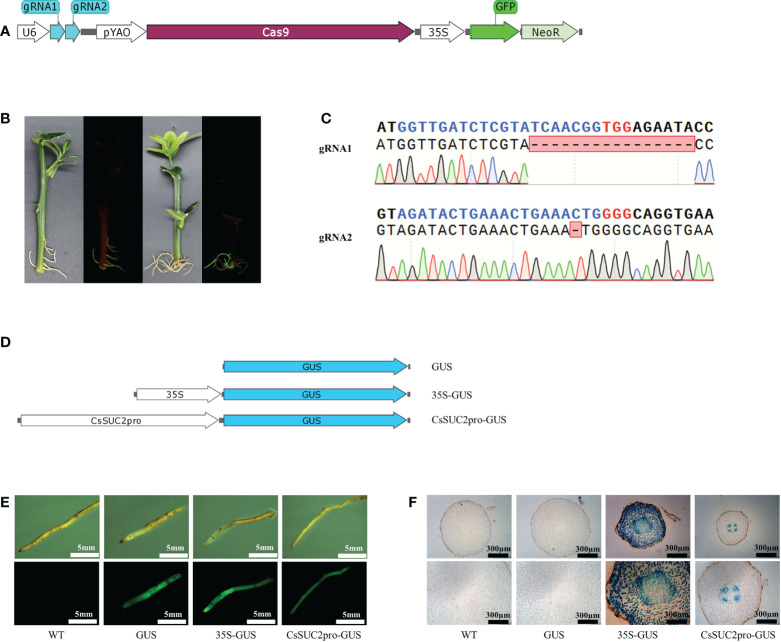
*Agrobacterium rhizogenes*-mediated genetic transformation complements “hard-to-get” transgenic materials and facilitates gene function analysis. **(A)** Schematic diagrams of *ACD2* gene-editing elements used in the study. gRNA1 and gRNA2 represent two gRNA scaffolds targeting *ACD2*. **(B)** Green fluorescence signal visualized by laser scanning confocal microscopy in transgenic tissues of citrus roots. **(C)** Alignment of the genome-edited sequence of *ACD2*-edited hairy roots with the gRNA-targeted loci. **(D)** Schematic diagrams of vectors containing different types of promoters. **(E)** Transgenic roots with GFP fluorescence were obtained after agroinfiltration using vectors containing different types of promoters. **(F)** GUS staining of transgenic roots.

Huanglongbing is among the most destructive citrus diseases worldwide and is caused by the phloem-limited bacterium *Candidatus* Liberibacter asiaticus (*C*Las). Citrus roots harbor the pathogens during *C*Las infection and are difficult to treat with bactericides. Our previous studies revealed that overexpression of *ACD2* in citrus promotes *C*Las multiplication (Pang et al., 2020). However, *ACD2*-edited or *ACD2*-silenced citrus roots have never been obtained using *A. tumefaciens*-mediated genetic transformation because *ACD2* family genes are involved in the regulation of cell death and all corresponding transgenic shoots ultimately die. In the present study, we successfully obtained *ACD2*-edited citrus roots using K599-mediated genetic transformation **(** **Figures 5A–C** **)**, indicating that this technology is advantageous for investigation of specific genes to obtain “hard-to-get” transgenic root tissue. In addition, *A. rhizogenes*-mediated citrus genetic transformation facilitates evaluation of tissue-specific promoters. When identifying exogenous genes that enhance resistance against a phloem-limited pathogen, a phloem-specific promoter is critical to reduce the impact of these genes on citrus biological traits (Dai et al., 2004; Tzean et al., 2020). In *Arabidopsis*, the companion cell-specific *AtSUC2* promoter has been widely used in gene function analysis of phloem-related genes (Paultre et al., 2016). In the present study, the homologous sequence of the *AtSUC2* promoter was identified in citrus and designated CsSUC2pro. Subsequently, we constructed three binary plasmids containing the *GFP* and *GUS* genes, of which CsSUC2pro, 35S, or no promoter was inserted at the 5′ end of GUS **(** **Figure 5D** **)**. Using K599-mediated genetic transformation, we obtained transgenic citrus hairy roots harboring these three plasmids **(** **Figure 5E** **)**. The results of GUS staining showed that all cells of 35S-GUS transgenic roots were stained blue, whereas only cells located in the phloem of CsSUC2-GUS transgenic roots were stained blue, and no cells of GUS (no promoter) transgenic roots were stained **(** **Figure 5F** **)**.

### *Agrobacterium rhizogenes*-mediated virus inoculation and its applications

**Figure 6:**
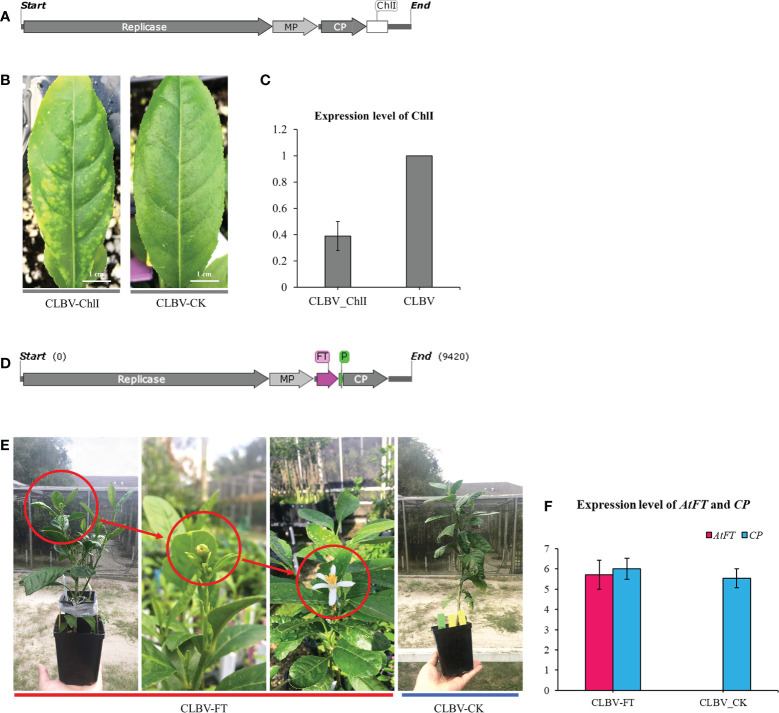
*Agrobacterium rhizogenes*-mediated citrus genetic transformation promotes the virus-induced application on citrus. **(A)** Schematic diagrams of the CLBV-ChlI vector. **(B)** Silencing phenotype of *Citrus medica* inoculated with wild-type CLBV (CLBV-CK) and CLBV-ChlI. **(C)** Relative accumulation of *ChlI* mRNA in *C*. *medica* inoculated with CLBV_CK or with CLBV_ChlI. **(D)** Schematic diagrams of the CLBV-FT vector generated by cloning the *FT* gene from *Arabidopsis thaliana*. **(E)** Flowering of a juvenile *C*. *medica* inoculated with the CLBV-FT. The juvenile *C*. *medica* inoculated with CLBV-CK did not flower. **(F)** Expression of *AtFT*.

Virus-induced gene silencing (VIGS), virus-mediated genome editing, or foreign gene overexpression technology have been widely used for gene function analysis in plants in recent years (Velázquez et al., 2016; Ellison et al., 2020). However, a viral vector cannot be transiently expressed in citrus leaves *via Agrobacterium* infiltration. Application of this technology to citrus is usually reliant on the use of tobacco (*Nicotiana* spp.) for virion enrichment. Given that K599-mediated genetic transformation of citrus is highly efficient, the feasibility of K599-mediated viral inoculation in citrus was examined. First, the *Citrus leaf blotch virus* (CLBV)-ChlI vector containing a partial sequence of *ChlI* (Magnesium chelatase subunit I) was constructed for *ChlI* silencing in citrus **(** **Figure 6A** **)**. *Citrus medica* explants were infiltrated with K599 harboring the CLBV-ChlI viral vector. Agroinfiltrated explants displayed photobleaching phenotypes in new leaves after 3 months **(** **Figure 6B** **)**. RT-qPCR results showed that the expression level of *ChlI* in CLBV-ChlI-transfected leaves was significantly downregulated, indicating that successful K599-mediated viral inoculation could be used for VIGS **(** **Figure 6C** **)**. Subsequently, the CLBV-FT vector **(** **Figure 6D** **)** containing the full-length sequence of the Arabidopsis thaliana *FLOWERING LOCUS* (*FT*) gene was constructed for *FT* overexpression in citrus based on a previously published method (Velázquez et al., 2016). The CLBV-FT vector was agroinfiltrated using 2-month-old *C. medica* seedlings, and the citrus infected with CLBV-FT flowered 9 months later **(** **Figure 6E** **)**. The RT-qPCR results showed that the expression level of *FT* in CLBV-FT-transfected *C. medica* was significantly upregulated **(** **Figure 6F** **)**.

## Discussion

Currently, *A. tumefaciens*, electroporation, particle bombardment, and RNA interference are used for citrus genetic transformation, but these methods are laborious, expensive, time-consuming, and inefficient. The limitation of these methods is largely due to their reliance on tissue culture, which requires an aseptic environment. Using *A. tumefaciens*-mediated genetic transformation as an example, the acquisition of transgenic citrus requires 4- to 5-week-old seedlings germinated on Murashige and Skoog medium, co-incubation of explants with *A*. *tumefaciens*, and shoot regeneration, and each step involves tissue culture under aseptic conditions (Orbović and Grosser, 2015). However, only few researchers have studied the genetic transformation of citrus bypassing tissue culture. With regard to *A. tumefaciens*, Chun-zhen Cheng developed an *in planta* genetic transformation approach for pomelo (*Citrus maxima*) and obtained transgenic plants using this method 3 months post-transformation (Zhang et al., 2017). On the other hand, virus-mediated genome editing in tobacco, Arabidopsis, and wheat has been reported (Ellison et al., 2020; Ghoshal et al., 2020; Ma et al., 2020; Luo et al., 2021). However, the aforementioned *in planta* genetic transformation technology requires 3–5 months to obtain transformants. In addition, virus-mediated genome-editing technology for woody plants has not been reported to date.

In recent years, the technology for obtaining transgenic roots using *A. rhizogenes* has been well developed in many herbaceous plants, such as soybean (Kereszt et al., 2007; Cao et al., 2009; Cheng et al., 2021), grain amaranth (Castellanos-Arévalo et al., 2020), rice (Raineri et al., 1990), and maize (Ishida et al., 2007). However, no report of *A. rhizogenes*-mediated endogenous gene editing bypassing tissue culture in citrus is available. In the present study, the genetic transformation procedure for citrus using *A. rhizogenes* bypassing tissue culture required only 2–4 weeks (*C. medica*) or 1–2 months (*C. limon* and citrange ‘Carrizo’). The explants used in this study are branches, which are highly convenient to obtain. Subsequently, we established a highly efficient, convenient, and cost-effective genome-editing technology system in citrus using this technology. In addition, the procedure can be used for efficient inoculation with viral vectors. Furthermore, hairy roots induced by *A. rhizogenes* often develop from single cells, which leads to a lower incidence of chimerism in transgenic roots (Roychowdhury et al., 2017). This phenomenon was confirmed using the technology in the present study. In our study, the transcriptome analysis of citrus callus induced by *A. rhizogenes* revealed that hormone (IAA) pathway was significantly triggered, which provide evidence to explain the highly efficient transformation rate in citrus. To improve the efficiency of verification of transgenic hairy roots, the binary vector (1380-Cas9-HA) used contains the GFP reporter gene. Thus, the hairy roots can be identified by hand-held excitation light detection of GFP, which is time- and labor-saving. The establishment of a highly efficient genetic transformation technology mediated by *A. rhizogenes* for multiple citrus species is of great importance for gene functional analysis in citrus.

In summary, we established a highly efficient genetic transformation technology bypassing tissue culture for citrus, which can be used for genome editing, gene overexpression, and virus-mediated gene function analysis. The advantages of this technology are as follows: (1) the explant used for transformation are citrus branches, which is convenient to obtain; (2) the transformation process does not involve tissue culture and thus is convenient to implement; (3) the process is time-saving (2–8 weeks); (4) the procedure is less labor demanding (as few branches are required); (5) a high frequency of positive transformants is obtained (~57%, *C. medica*); (6) gene transformation or genome editing are achieved with high efficiency (**Table S10**). The problems that may be encountered during the experiment and the corresponding solutions were listed in **Table S11**. We anticipate that by removing the high cost, heavy workload, long experimental period, and other technical obstacles, this genetic transformation technology will be a valuable tool for routine investigation of endogenous and exogenous genes in citrus.

## Data availability statement

The transcriptome data presented in the study are deposited in the NCBI repository, accession number PRJNA800116.

## Author contributions

Conceptualization, HM, SD, XS; writing—original draft preparation, HM; writing—review and editing, SD, XS, FG, NW; supervision, HM, SD, XS, JL; project administration, HM, XM; transcriptome analysis, YG; experiment, XM, MW, NL, SH, ML; plants maintenance, XM, HX, KX, MW; funding acquisition, JL, ML. All authors contributed to the article and approved the submitted version.

## Funding

This work was supported by the National Natural Science Foundation of China (32202427 and 32002021); the Key Project for New Agricultural Cultivar Breeding in Zhejiang Province, China (2021C02066-1); The Major Science and Technology R& D Program of Jiangxi Province (20194ABC28007).

## Acknowledgments

We thank Honghong Deng, Fabieli Irizarry, Ming Huang, Chen Ling, Jiaying Fang, Hujing Wang, and Qibin Yu for assistance with plant materials and laboratory activities.

## Conflict of interest

JL was employed by Natural Medicine Institute of Zhejiang YangShengTang Co., LTD.

The remaining authors declare that the research was conducted in the absence of any commercial or financial relationships that could be construed as a potential conflict of interest.

## Publisher’s note

All claims expressed in this article are solely those of the authors and do not necessarily represent those of their affiliated organizations, or those of the publisher, the editors and the reviewers. Any product that may be evaluated in this article, or claim that may be made by its manufacturer, is not guaranteed or endorsed by the publisher.
