# Supplementary material for: Highly efficient hairy root genetic transformation and applications in citrus
Source: Front Plant Sci. 2022 Oct 27;13:1039094. doi: 10.3389/fpls.2022.1039094 (PMC9647159; doi:10.3389/fpls.2022.1039094)
Supplement: Supplementary file 2 [file Presentation_1.pptx]

## Slide 1
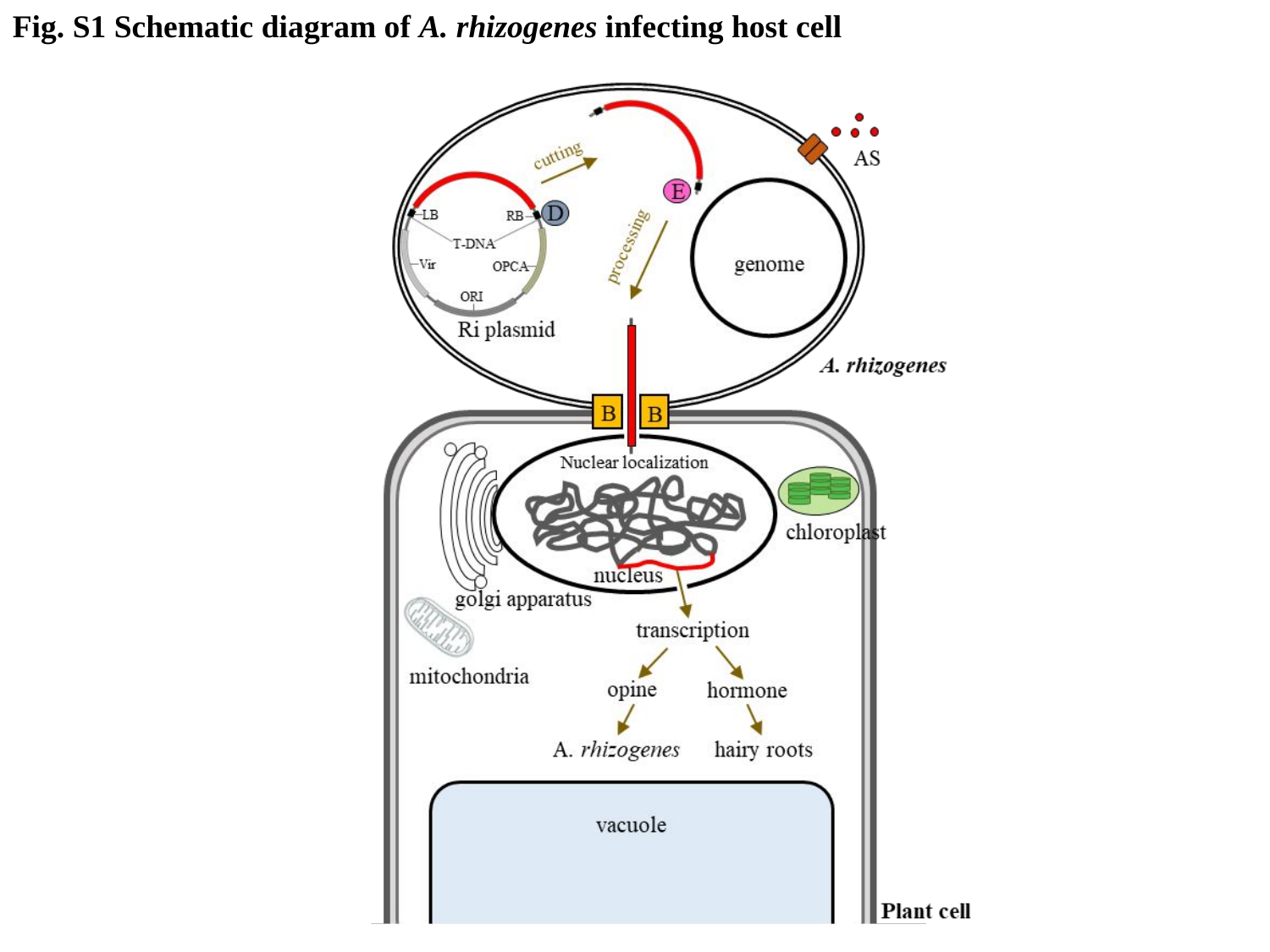

Fig. S1 Schematic diagram of A. rhizogenes infecting host cell

## Slide 2
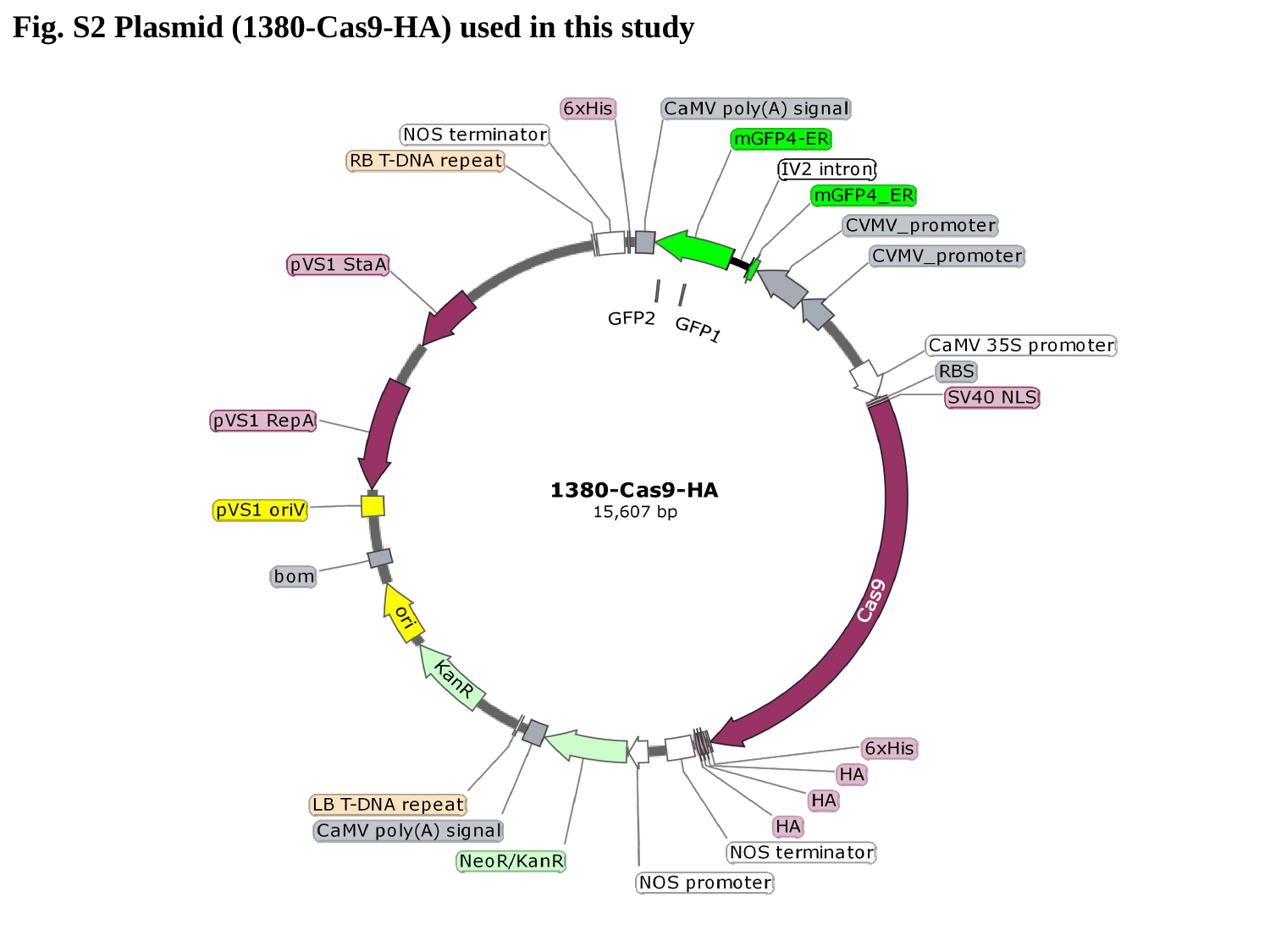

Fig. S2 Plasmid (1380-Cas9-HA) used in this study

## Slide 3
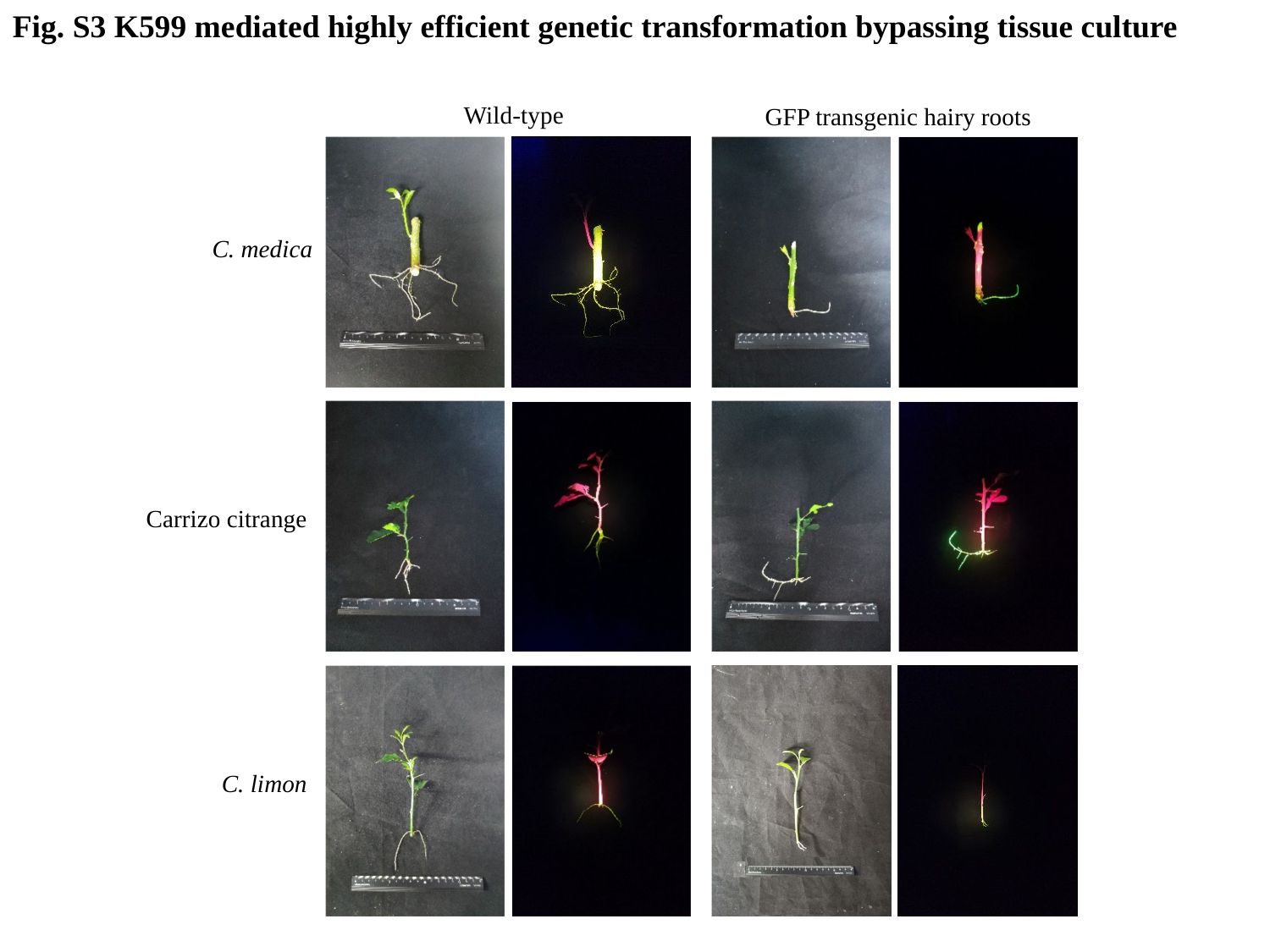

Fig. S3 K599 mediated highly efficient genetic transformation bypassing tissue culture
Wild-type
GFP transgenic hairy roots
C. medica
Carrizo citrange
C. limon

## Slide 4
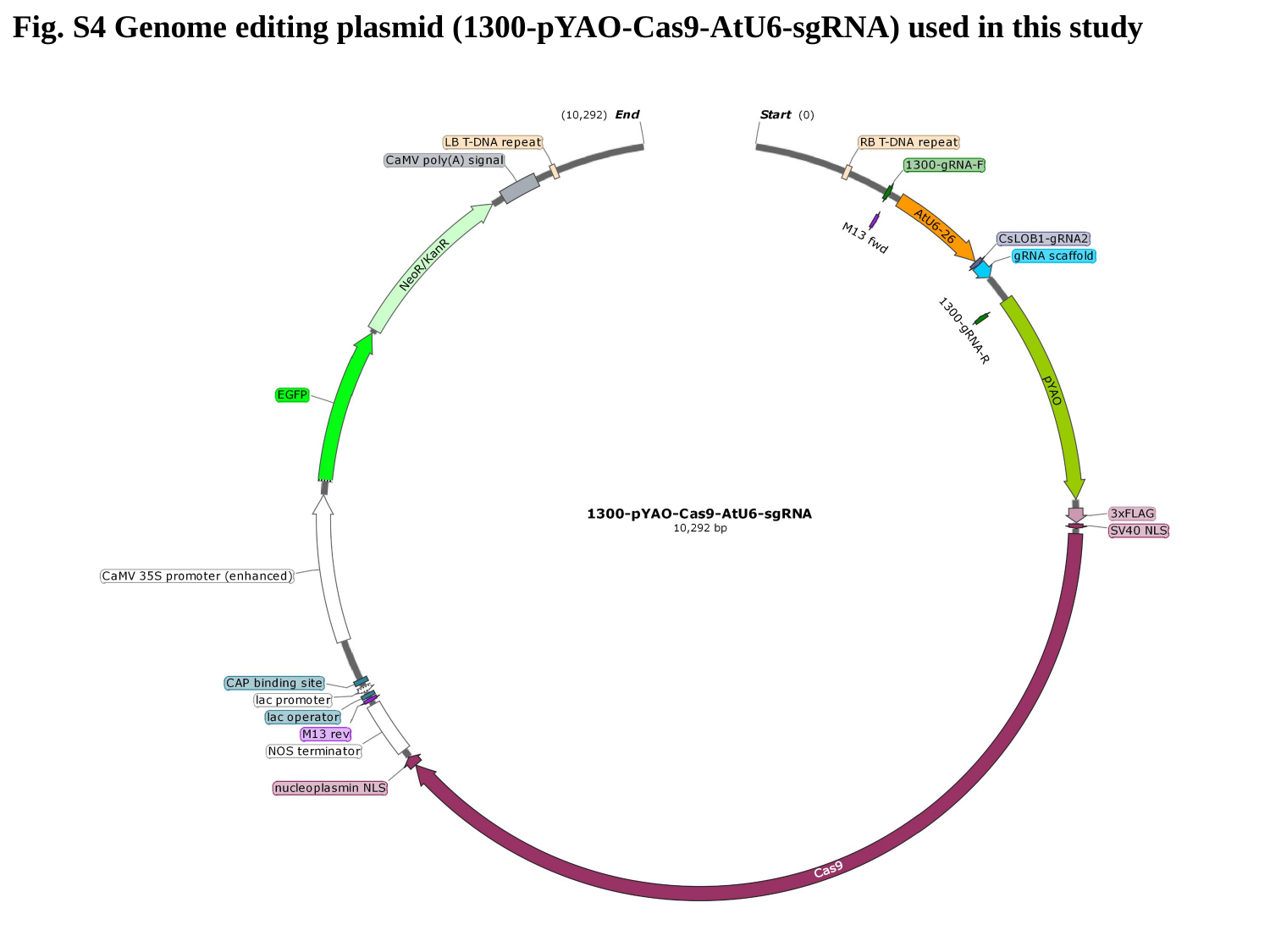

Fig. S4 Genome editing plasmid (1300-pYAO-Cas9-AtU6-sgRNA) used in this study

## Slide 5
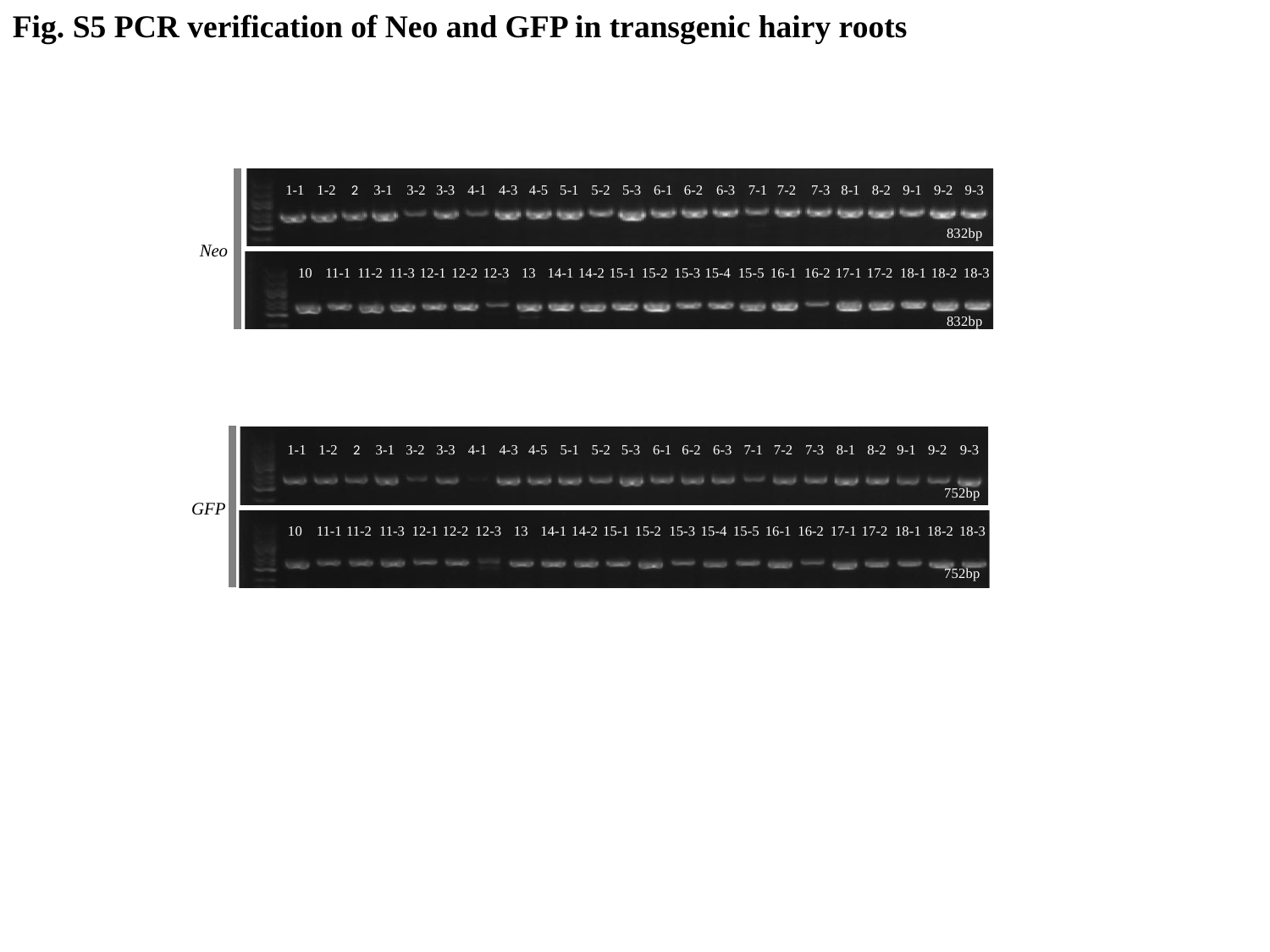

Fig. S5 PCR verification of Neo and GFP in transgenic hairy roots
1-1
1-2
2
3-1
3-2
3-3
4-1
4-3
4-5
5-1
5-2
5-3
6-1
6-2
6-3
7-1
7-2
7-3
8-1
8-2
9-1
9-2
9-3
832bp
Neo
14-1
10
11-1
11-2
11-3
12-1
12-2
12-3
13
14-2
15-1
15-2
15-3
15-4
15-5
16-1
16-2
17-1
17-2
18-1
18-2
18-3
832bp
1-1
1-2
2
3-1
3-2
3-3
4-1
4-3
4-5
5-1
5-2
5-3
6-1
6-2
6-3
7-1
7-2
7-3
8-1
8-2
9-1
9-2
9-3
752bp
GFP
10
11-2
11-3
12-1
12-2
12-3
13
15-1
15-2
15-3
15-4
15-5
16-1
16-2
17-1
17-2
18-1
18-2
18-3
11-1
14-1
14-2
752bp

## Slide 6
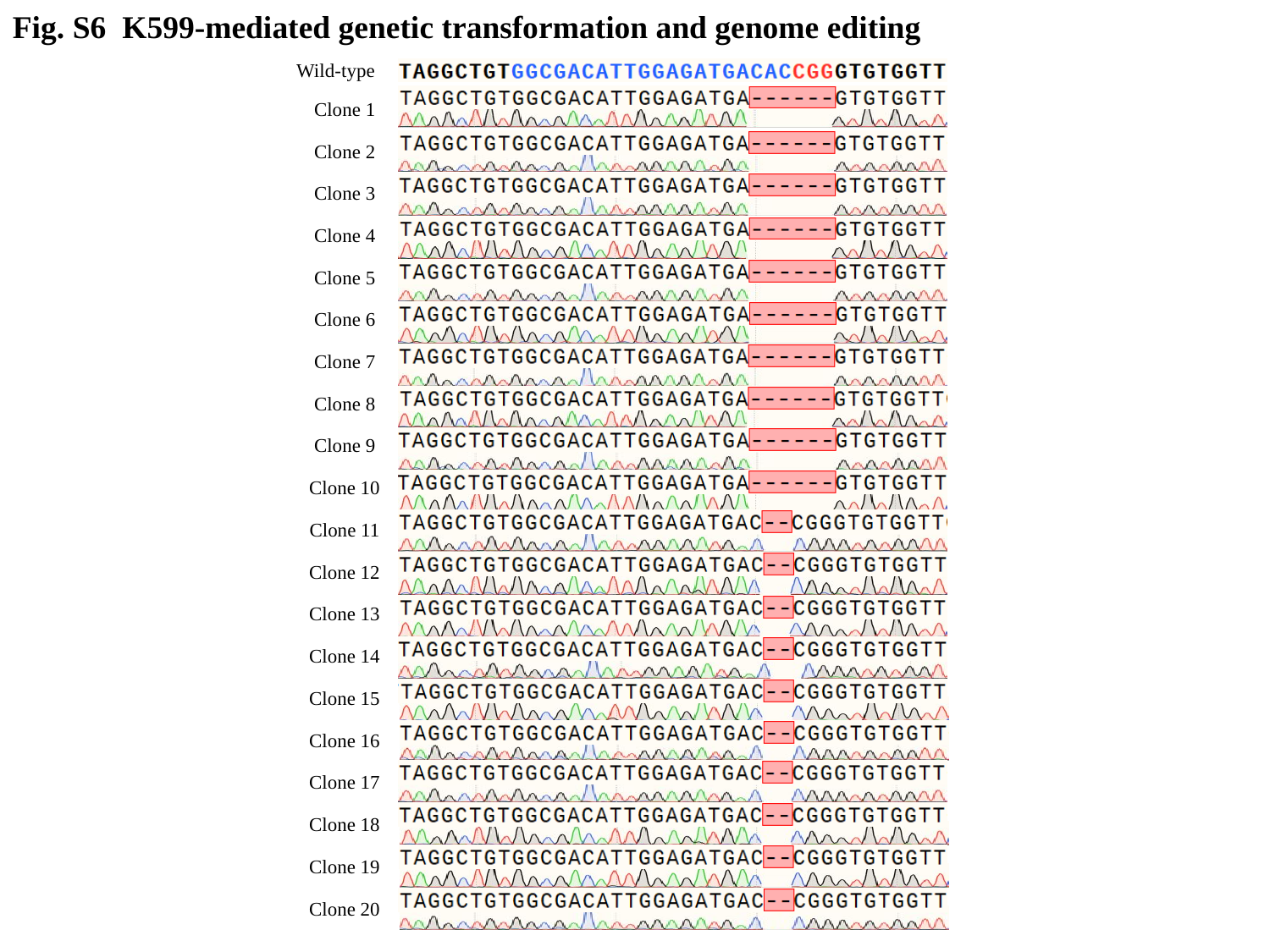

Fig. S6 K599-mediated genetic transformation and genome editing
Wild-type
Clone 1
Clone 2
Clone 3
Clone 4
Clone 5
Clone 6
Clone 7
Clone 8
Clone 9
Clone 10
Clone 11
Clone 12
Clone 13
Clone 14
Clone 15
Clone 16
Clone 17
Clone 18
Clone 19
Clone 20

## Slide 7
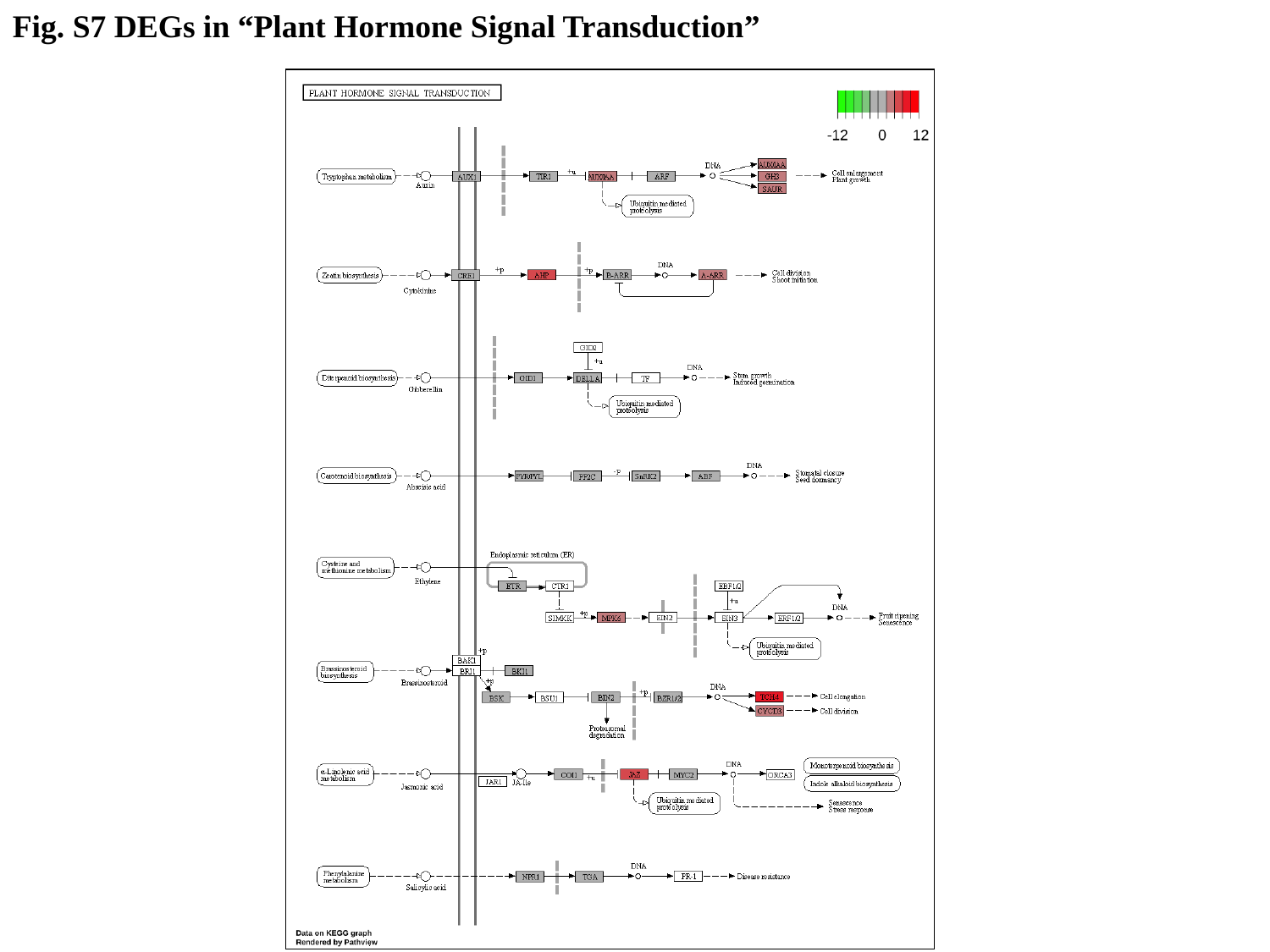

Fig. S7 DEGs in “Plant Hormone Signal Transduction”

## Slide 8
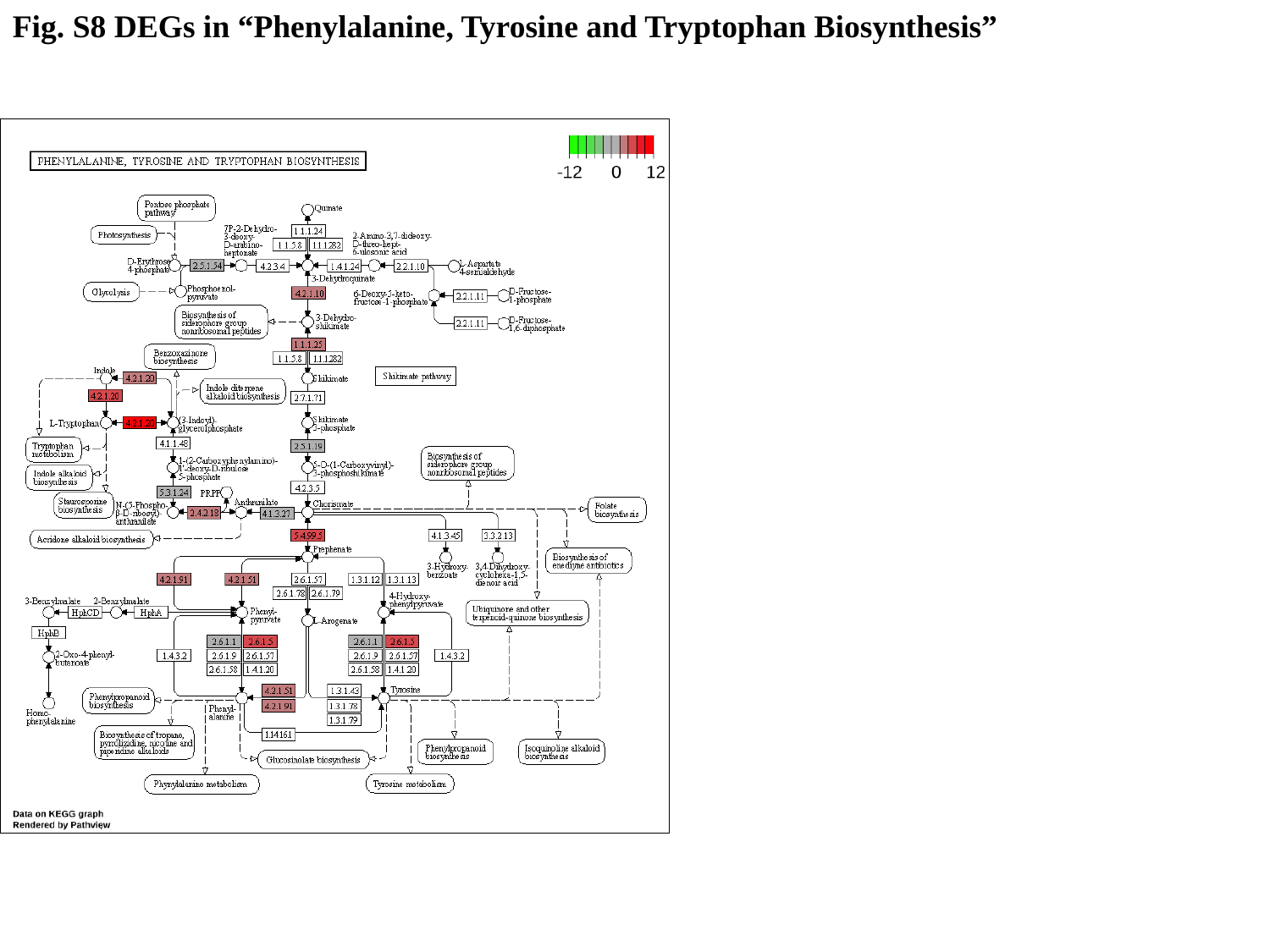

Fig. S8 DEGs in “Phenylalanine, Tyrosine and Tryptophan Biosynthesis”

## Slide 9
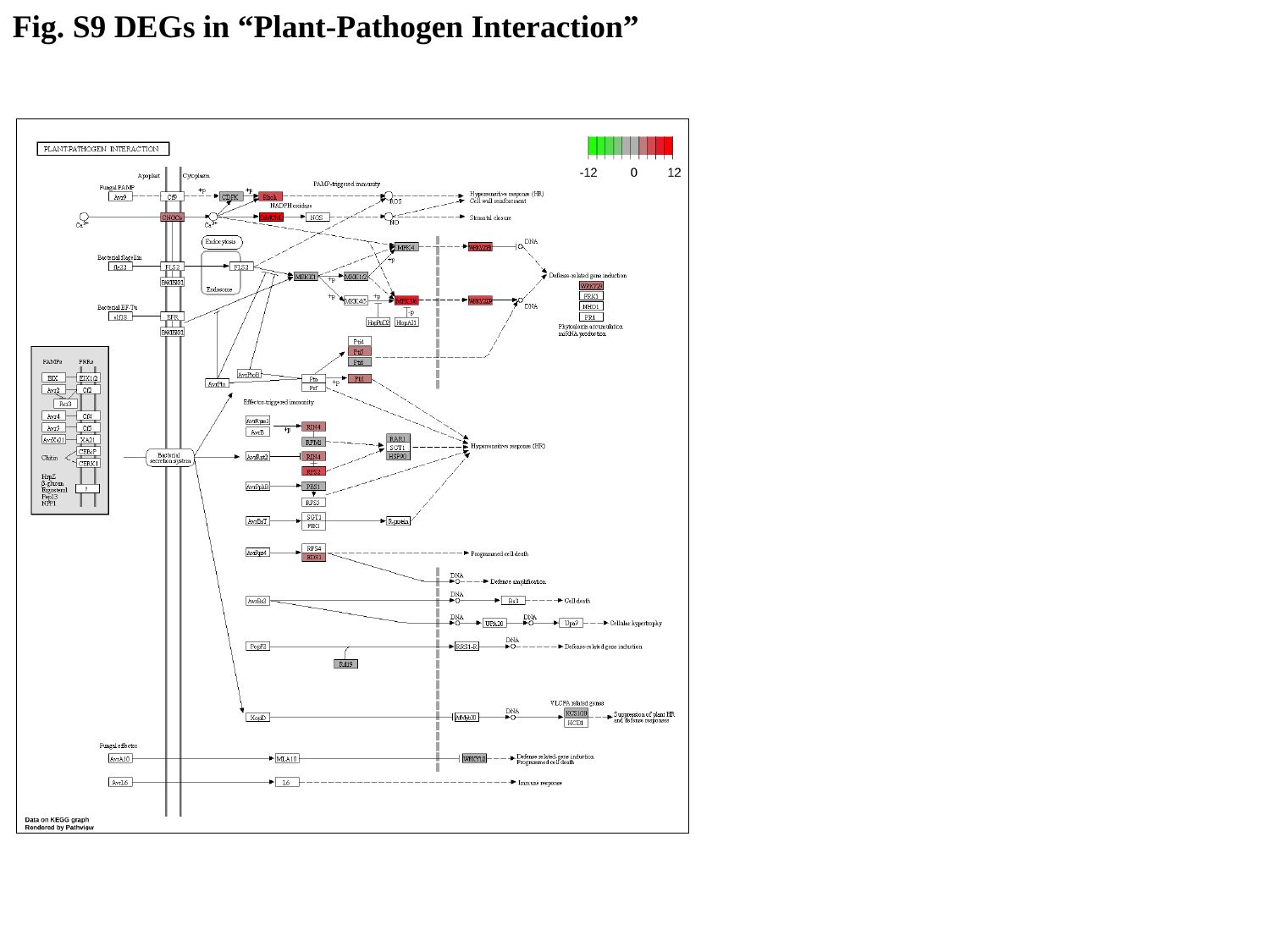

Fig. S9 DEGs in “Plant-Pathogen Interaction”

## Slide 10
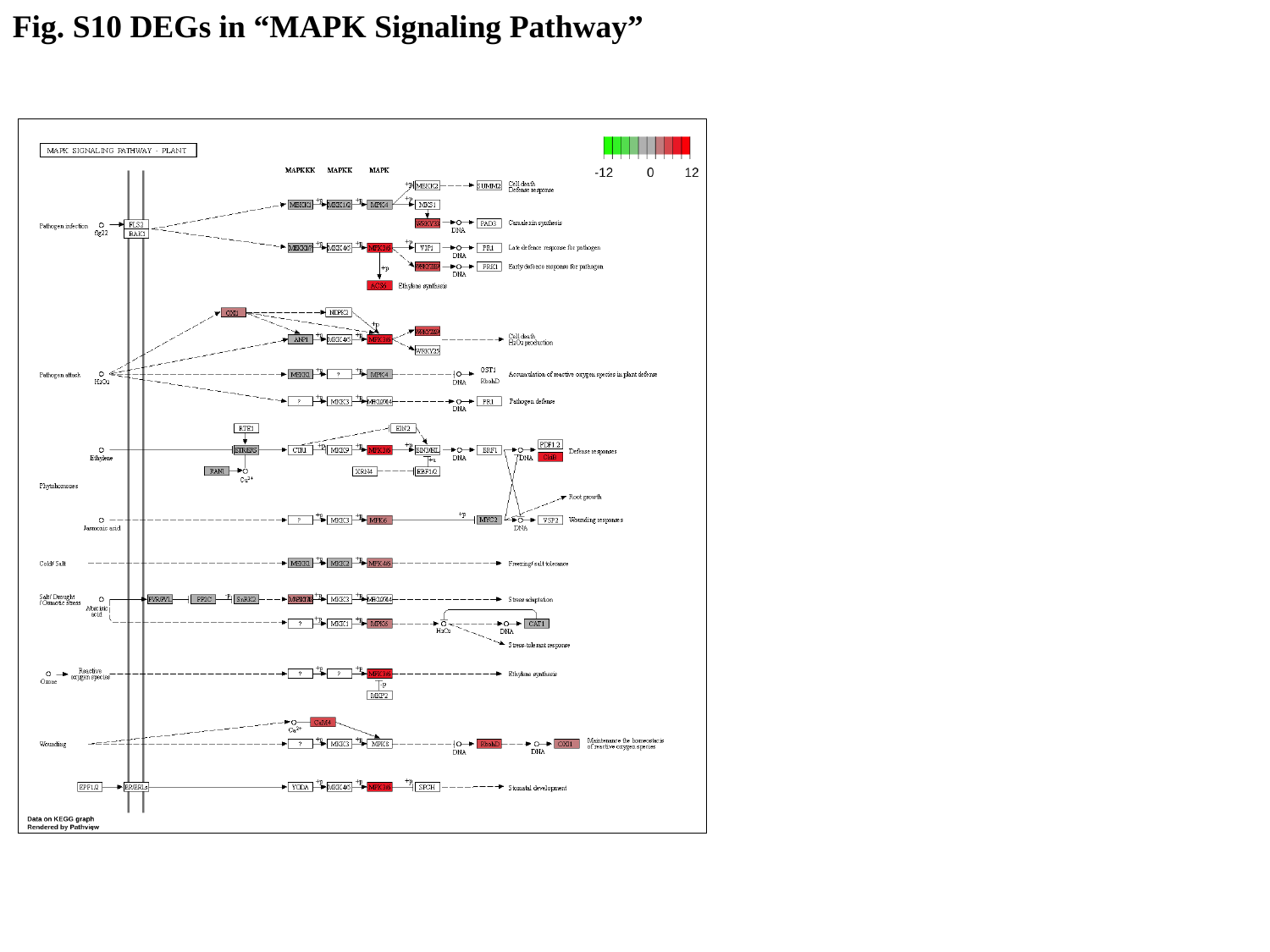

Fig. S10 DEGs in “MAPK Signaling Pathway”

## Slide 11
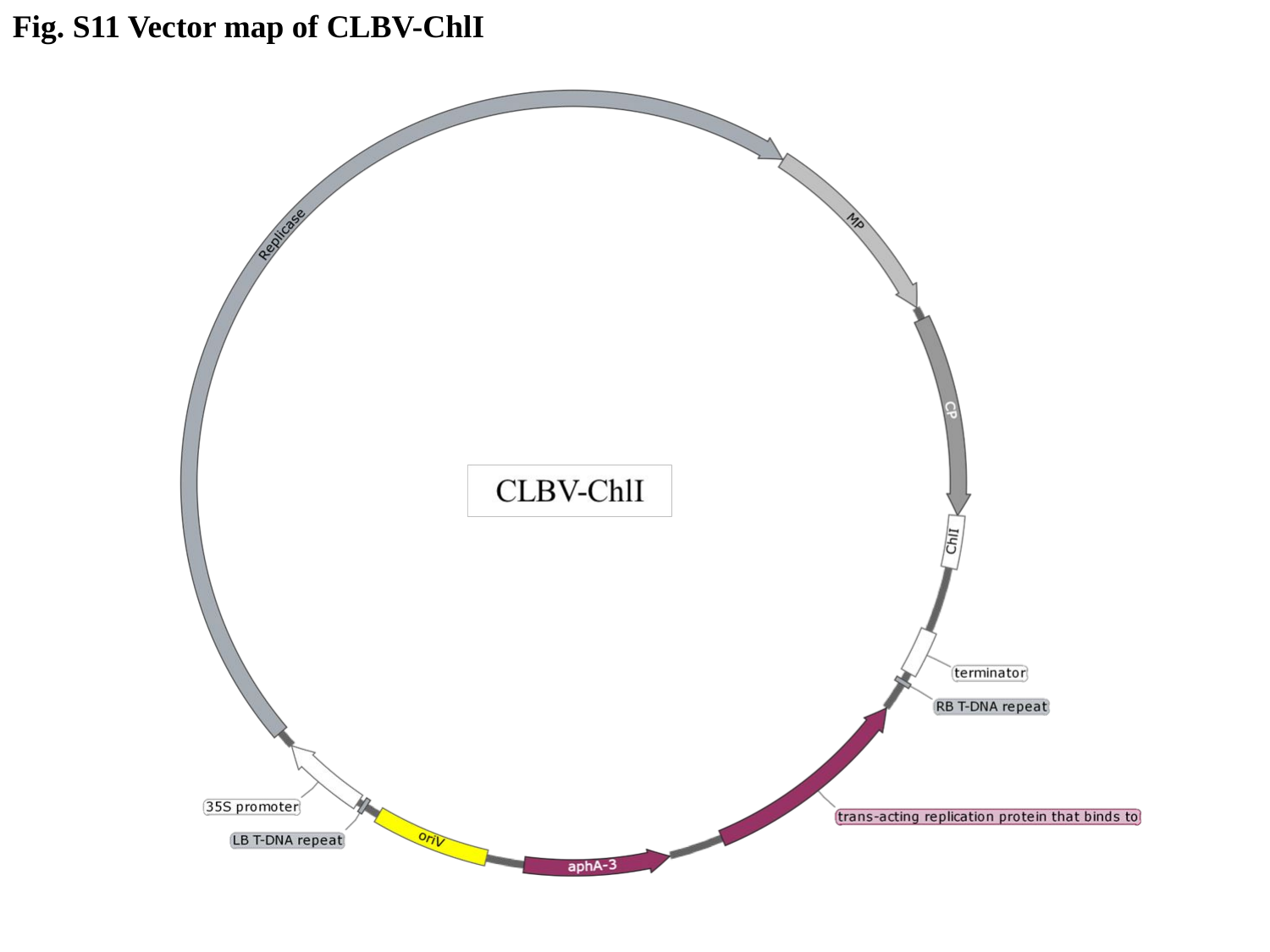

Fig. S11 Vector map of CLBV-ChlI
